# Health and Behavioral Characteristics of Metaverse Users in Japan: Metaverse Health Survey 2025

**DOI:** 10.7759/cureus.85590

**Published:** 2025-06-09

**Authors:** Ryo Momosaki, Kazuma Tora, Yuka Shirai, Yoko Hasegawa, Kenta Ushida, Ryota Sakamoto, Hiroki Funao

**Affiliations:** 1 Department of Rehabilitation Medicine, Graduate School of Medicine, Mie University, Tsu, JPN; 2 Department of Rehabilitation, Akiyama Clinic, Takamatsu, JPN; 3 Clinical Nutrition Unit, Hamamatsu University Hospital, Hamamatsu, JPN; 4 Department of Rehabilitation, Mie University Hospital, Tsu, JPN; 5 Department of Medical Informatics, Mie University Hospital, Tsu, JPN; 6 Department of Practical Nursing, Graduate School of Medicine, Mie University, Tsu, JPN; 7 Department of Molecular Pathobiology and Cell Adhesion Biology, Mie University Graduate School of Medicine, Tsu, JPN

**Keywords:** cybersickness, diet, exercise, health literacy, mental health, metaverse, nutritional status, physical activity, sleep, virtual reality

## Abstract

Background

The metaverse has rapidly evolved into a significant virtual ecosystem, attracting millions of users worldwide. While it offers opportunities for social connectivity and enhanced mental well-being, prolonged metaverse engagement raises concerns regarding physical inactivity, unbalanced dietary habits, and sleep health. This study aimed to clarify the health status of metaverse users.

Methodology

An anonymous online survey was conducted from April to May 2025, targeting metaverse users aged ≥16 years, primarily residing in Japan. The survey covered domains such as physical activity, dietary habits, mental health, sleep quality, and health literacy, using standardized scales and questionnaires. Descriptive statistics were used to summarize user health profiles and behaviors.

Results

Among the 417 participants, the majority were males and in their 20s and 30s. Over half reported sedentary behavior of ≥8 hours per day. Dietary diversity was limited, with 177 (55.1%) of participants scoring ≤2 out of 10 on the dietary variety score. Short sleep duration (<6 hours/night) was observed in 110 (26.4%) of participants, and 75 (18.0%) reported worsened sleep quality since starting metaverse use. In contrast, 158 (37.9%) experienced improvements in mental health. Physical activity was also reported: 97 (23.3%) engaged in metaverse-based exercise routines, and 81 (19.4%) regularly chose a standing posture during use. Most participants were in the contemplation stage regarding changes to diet and exercise behaviors. Additionally, 283 (67.9%) expressed interest in accessing medical consultations within the metaverse.

Conclusions

While various health issues, such as insufficient physical activity, unbalanced diets, and poor sleep, were observed, the presence of users who stand, exercise, and report mental health benefits within the metaverse was also evident. The integration of health promotion strategies into the metaverse presents a promising opportunity to support behavioral change. Future efforts should focus on developing and implementing frameworks that facilitate health through enjoyable and immersive experiences in the metaverse.

## Introduction

In recent years, advancements in augmented reality, cross-reality technologies, and computing have enabled prolonged gaming experiences and the hosting of events within virtual environments such as the metaverse. The metaverse is gaining attention as a technology that extends the real world and introduces new possibilities. With its increasing popularity, some metaverse platforms now report user bases ranging from 50 to 200 million worldwide [1,2]. The metaverse is not merely a virtual space but rather an ecosystem composed of communities formed by users who regularly log in and engage in daily activities within it [3]. This has given rise to *metaverse residents*, individuals who spend extended periods immersed in these environments.

However, concerns have been raised regarding potential health risks associated with prolonged metaverse use [4]. Extended engagement in online gaming, including within the metaverse, has been linked to increased sedentary behavior and reduced cardiorespiratory fitness, suggesting that metaverse residents may be at risk for physical function decline [5]. In addition, extended screen time spent consuming gaming content has been linked to unhealthy patterns [6], reduced sleep duration, and delayed bedtimes [7]. A preliminary survey conducted during an academic conference held in the metaverse revealed that many participants remained logged in and seated for extended periods, raising health-related concerns [8]. Despite this, comprehensive information on the health status of metaverse users remains limited.

Many metaverse residents are highly creative and capable individuals; therefore, a deterioration in their health could represent a significant societal loss. Clarifying the health status of metaverse users is essential to establishing foundational knowledge for promoting healthier lifestyles within virtual environments. This cross-sectional, anonymous online survey aims to (1) profile key modifiable health behaviors - physical activity, sedentary time, diet quality, sleep, and alcohol use - and (2) describe related physical and mental health outcomes among adult metaverse users.

## Materials and methods

An anonymous online survey was conducted between April 5 and May 6, 2025. Eligible participants were individuals aged 16 or older who had accessed any metaverse platform (e.g., VRChat [VRChat, Inc., San Francisco, CA], cluster, Neos VR, Resonite, Spatial, My Vket, DOOR, Rec Room, Roblox) at least once in the preceding month. Participants could use any device to access the metaverse, including head-mounted displays, computers, smartphones, or tablets. No other exclusion criteria were set, however, individuals who are unable to complete the questionnaire due to cognitive impairment, those with severe physical limitations, or those who do not have access to the digital devices necessary to participate in the metaverse are not expected to participate. Recruitment was carried out via posts on a social media account with approximately 1,900 followers on platform X. The X account is the personal account of the first author, and it contains many posts related to the metaverse and virtual reality. Followers are mainly composed of metaverse users and members of the virtual reality community, however, they may not adequately represent the broad population of metaverse users. In addition, the sampling method used in this study is self-selection sampling. Since participants voluntarily gathered based on their own interests, there is a possibility of bias in the respondents' motivation, interests, and characteristics. We also posted an X advertisement in the same X account for research participation, which was viewed by approximately 50,000 people. The details of the algorithm used to display the advertisement are not clear, however, the advertisement was displayed to users with attributes similar to those of the account that posted. By displaying the advertisement, we were able to reach users who normally would not have any connection with the author's account, which may have reduced the imbalance of respondents. Informed consent was obtained through an explanatory document attached to the Google Form survey. Those who consented completed a five-minute questionnaire. The explanatory material was provided in Japanese and English, with Google Translate functionality available to facilitate international responses. Participants would have the opportunity to win Amazon gift cards (5,000 yen for one person and 1,000 yen for five people) at random. There are no previous studies on the health status of metaverse users, and we were unable to obtain the information necessary for sample size calculation. Therefore, we decided to collect as many samples as possible within the available range. This study was approved by the Clinical Research Ethics Review Committee of Mie University Hospital (H2025-006).

Survey items

Survey questions were designed with a focus on physical activity, dietary and nutritional status, and mental health. The following categories were included:

(1) Background information: Age, sex, geographic location, employment status, and number of cohabitants

(2) Metaverse usage: Primary platforms used, primary access device, duration of metaverse use, usage frequency, average session duration, and number of friends within the metaverse

(3) General health status: Current health condition, health-related concerns, smoking habits

(4) Physical activity and physical function: Exercise habits (including in-metaverse activity), daily sedentary time, typical posture during metaverse use, weekly frequency of going out, perceived changes in physical activity, fitness, muscle strength, and visual acuity since metaverse use began, body pain, experience of cybersickness, weekly moderate-to-vigorous physical activity, and behavior change stage regarding exercise

(5) Dietary and nutritional status: Body mass index (BMI), weight change since beginning use of the metaverse, dietary balance (measured using a dietary variety score), alcohol consumption changes, breakfast consumption, post-dinner snacking habits, and behavior change stage related to diet

(6) Mental health and sleep-related information: Current mental health issues, changes in mental health after beginning metaverse use, negative impacts of metaverse use on daily life, sleeping while wearing a head-mounted display, sleep duration and timing, and changes in sleep quality

(7) Other factors: Health literacy, interest in consulting healthcare professionals within the metaverse

Moderate-to-vigorous physical activity was assessed using the Physical Activity Vital Sign [9], calculated by multiplying responses to two questions: “On how many days per week do you engage in moderate-to-vigorous physical activity (e.g., brisk walking)?” and “On average, how many minutes per session?” This method, while simple and not without limitations, was chosen to minimize respondent burden.

Dietary balance was assessed using the dietary variety score [10]. Participants reported the frequency of consumption for 10 food groups: meat, fish, eggs, milk and dairy products, soybean products, green and yellow vegetables, fruits, potatoes, oils, and seaweed. Groups that consumed *almost every day* scored 1 point; all other responses scored 0 points. A score of ≥7 was considered ideal.

Behavioral stages for exercise and dietary habits were evaluated using standard questionnaire items from Japanese health checkups [11] based on the question, “Are you going to start, or have you started lifestyle modification lifestyle habits, such as exercise and diet?” Responses were categorized as follows: (1) Not planning to start; (2) planning to start in the future (e.g., within six months); (3) planning to start soon (e.g., within a month) or just started; (4) started within the past six months, and (5) started more than six months ago. These responses correspond to the five stages of behavior change: precontemplation, contemplation, preparation, action, and maintenance.

The presence or absence of current mental health issues was assessed using a single-item measure of self-rated mental health [12]. Participants responded *yes* or *no* to the question: "In general, would you say your mental health is good?" Those answering *no* were categorized as having mental health issues. While this question has demonstrated clinical utility, restricting responses to *yes* or *no* may have led to a loss of subtle differences or gradations in participants' answers. For mental health-related questions other than this, we did not use existing validated measurement scales. Regarding interpersonal stress in the metaverse, respondents were asked, “Do you feel stress related to interpersonal relationships in the metaverse?” and answered ‘yes’ or “no.” Regarding changes in mental health after beginning metaverse use, we asked, “Have you experienced any changes in your mental health after beginning metaverse use?” and asked respondents to select “improved,” *worsened*, or *no change*. Regarding the negative impact of metaverse use on daily life, respondents answered *yes* or *no* to the question: “Does the use of the metaverse have a negative impact on your daily life?” Regarding changes in sleep quality, respondents answered *improved*, *worsened*, or *no change* to the question: “Has your sleep quality changed compared to before you started using the metaverse?” Many questions have not been verified for validity, so we have mentioned this in the limitations.

Health literacy was assessed using the Communicative and Critical Health Literacy Scale [13]. Participants rated their agreement (on a five-point Likert scale) with the following statements: (1) I can obtain health-related information from various sources; (2) I can extract the necessary information; (3) I can understand and communicate the information; (4) I can assess the reliability of the information; and (5) I can make decisions based on the information. The final score was calculated as the mean of the five items (range: 1-5): 1. (strongly disagree), 2. (disagree a little), 3. (neither disagree nor agree), 4. (agree a little), and 5. (strongly agree). Participants were classified into three groups based on their scores: low (1.0-3.0), medium (3.1-3.9), and high health literacy (4.0-5.0).

Descriptive statistics were used to analyze the survey data. Frequencies and percentages were reported for categorical variables. For continuous variables, the mean and standard deviation were reported if normally distributed, and the median and interquartile range were reported if not. Normality was visually determined from the histogram. Missing values below 1% were imputed using the missForest method via the R statistical package [14]. As this study was descriptive in nature, no statistical hypothesis testing was conducted [15].

## Results

Responses were received from 417 participants, with less than 1% missing data across all variables. Background characteristics are detailed in Table 1. The majority of participants were aged 30-39 years at 158 (37.9%), followed by those aged 20-29 at 126 (30.2%). Most respondents were male at 291 (69.8%), lived in Japan at 401 (96.2%), were employed at 353 (84.7%), and lived alone at 136 (32.6%).

**Table 1:** Sociodemographic characteristics of participants: age, sex, living location, employment, and living arrangement (n = 417)

Sociodemographic characteristics	*n* (%)
Age (year)	
<20	25 (6.0)
20-29	126 (30.2)
30-39	158 (37.9)
40-49	62 (14.8)
50-59	33 (7.9)
>59	13 (3.0)
Sex	
Male	291 (69.8)
Female	111 (26.6)
Other	15 (3.6)
Living location	
Japan	401 (96.2)
Outside Japan	16 (3.8)
Working	353 (84.7)
Living alone	136 (32.6)

Metaverse usage was primarily via VRChat at 253 (60.7%) and the cluster at 146 (35.0%) (Table 2). The most common access device was a head-mounted display with PC at 250 (60.0%), followed by PC only at 105 (25.2%). Over half of the respondents had been using the metaverse for more than two years, with most engaging at least every other day, and more than 50% playing for over three hours per session.

**Table 2:** Metaverse usage characteristics: platform, device, duration, frequency, session time, and number of friends.

Metaverse usage characteristics	*n* (%)
Most commonly used metaverse platforms	
VRChat	253 (60.7)
Cluster	146 (35.0)
Other	18 (4.3)
The most commonly used device for the metaverse	
Head-mounted displays and PCs	250 (60.0)
PCs	105 (25.2)
Head-mounted displays	33 (7.9)
Smartphones or tablets	29 (6.9)
Duration of metaverse use	
Less than 1 year	84 (20.1)
About 1 year	58 (13.9)
About 2 years	84 (20.1)
About 3 years	84 (20.1)
About 4 years	60 (14.4)
Over 5 years	47 (11.3)
Frequency of metaverse use	
Every day	146 (35.0)
Not every day, at least every other day	83 (20.0)
Less than every other day	188 (45.0)
Hours spent metaverse playing per session	
Less than 1 hour	21 (5.0)
About 1 hour	34 (8.2)
About 2 hours	134 (32.1)
About 3 hours	109 (26.1)
Over 4 hours	119 (28.5)
Number of friends in the metaverse	
<100	179 (42.9)
100-500	164 (39.3)
>500	74 (17.7)

Table 3 shows health, physical activity, and function data. Of the respondents, 166 (39.8%) reported current health problems, and 249 (59.7%) expressed concern about their future health. Smokers comprised 61 (14.6%) of the respondents. While 223 (53.5%) had regular exercise habits, 97 (23.3%) exercised within the metaverse. By gender, 175 men (60.1%) and 42 women (37.8%) had regular exercise habits. Over half of the respondents reported sitting for eight or more hours daily, and 81 (19.4%) reported standing while using the metaverse. The most common response regarding going out was *5-6 days a week* at 197 (47.2%); however, 79 (18.9%) of respondents experienced reduced physical activity, 99 (23.7%) reported lower physical fitness, 84 (20.1%) noted reduced muscle strength, and 142 (34.1%) reported physical pain. In addition, cybersickness was experienced by 142 (34.1%), while vision worsened in 76 (18.2%) and improved in 30 (7.2%). A high proportion (36.5%) reported zero minutes of moderate-to-vigorous physical activity, with a median and interquartile range of 40 (0, 170) minutes. The most common stage of exercise behavior was *Contemplation* at 162 (38.8%).

**Table 3:** General health status, physical activity, and physical function associated with metaverse use.

	*n* (%)
Current health problems	166 (39.8)
Concern about future health	249 (59.7)
Smoking habits	61 (14.6)
Exercise habits	223 (53.5)
Exercise habits in the metaverse	97 (23.3)
Daily sedentary time	
Less than 4 hours	78 (18.6)
5-7 hours	117 (28.1)
8-9 hours	87 (20.9)
10-11 hours	70 (16.8)
12 hours or more	65 (15.6)
Posture during metaverse use	
Sitting	300 (71.9)
Standing	19.4 (19.4)
Lying down	36 (8.6)
Frequency of going out per week	
7 days	73 (17.5)
5-6 days	197 (47.2)
2-4 days	119 (28.6)
0-1 day	28 (6.8)
Decreased physical activity since starting metaverse use	79 (18.9)
Decrease in physical fitness since starting metaverse use	99 (23.7)
Muscle weakness since starting metaverse use	84 (20.1)
Physical pain since starting metaverse use	142 (34.1)
Experience of cybersickness in the metaverse	142 (34.1)
Changes in visual acuity since starting metaverse use	
Improved	30 (7.2)
Decreased	76 (18.2)
No change	311 (80.3)
Moderate-to-vigorous physical activity per week (minutes)	
0	152 (36.5)
1-74	105 (25.2)
75-149	48 (11.5)
150-299	51 (12.2)
300 or more	61 (14.6)
Stage of change in exercise behavior	
Precontemplation	52 (12.5)
Contemplation	162 (38.8)
Preparation	76 (18.2)
Action	58 (13.9)
Maintenance	69 (16.5)

Dietary and nutritional status (Table 4) showed that 268 (64.3%) had a normal BMI, with 75 (18.0%) overweight and 36 (8.6%) obese (mean BMI: 23.2 ± 4.7). Approximately 77 (18.4%) of participants had gained over 5 kg postmetaverse adoption. The dietary variety score averaged 2.4 (±1.9), with 230 (55.1%) of participants scoring 2. Postmetaverse, 87 (20.9%) of respondents reported an increase in alcohol consumption. Breakfast skipping was reported by 230 (55.1%), and 306 (73.4%) snacked after dinner. The most common stage of dietary behavior was *Contemplation* at 181 (43.3%).

**Table 4:** Nutritional status and dietary behaviors associated with metaverse use.

	*n* (%)
Body mass index (kg/m^2^)	
Underweight <18.5	38 (9.1)
Normal weight 18.5-24.9	268 (64.3)
Overweight 25-29.9	75 (18.0)
Obesity 30.0-34.9	36 (8.6)
Weight gain ≥5 kg since starting metaverse use	77 (18.4)
Dietary Variety Score	
0-2 (low variety)	177 (55.1)
3-5 (moderate variety)	122 (38.0)
6-10 (high variety)	22 (6.9)
Increased alcohol consumption since starting metaverse use	87 (20.9)
Sometimes skips breakfast	230 (55.1)
Sometimes snacks after dinner	306 (73.4)
Stages of change in dietary behavior	
Precontemplation	100 (24.0)
Contemplation	181 (43.3)
Preparation	57 (13.7)
Action	33 (7.9)
Maintenance	46 (11.0)

Mental health and sleep-related information showed that 195 (46.8%) of respondents reported current mental health issues, and 91 (21.8%) experienced interpersonal stress within the metaverse (Table 5). However, 158 (37.9%) reported mental health improvement post-metaverse, while 96 (23.0%) noted negative effects on daily life. 76 (22.3%) of participants reported sleeping while wearing a head-mounted display, while 110 (26.4%) reported sleeping less than 6 hours per night, and 75 (18.0%) experienced a decline in sleep quality. Furthermore, late bedtimes were common, with 272 (65.2%) of the respondents going to bed after 1:00 a.m.

**Table 5:** Mental health and sleep patterns associated with metaverse use.

	*n* (%)
Currently experiencing mental health problems	195 (46.8)
Interpersonal stress in the metaverse	91 (21.8)
Mental health changes since starting metaverse use	
Improved	158 (37.9)
Decreased	37 (8.9)
No change	222 (53.2)
Negative impact of metaverse play on daily life	96 (23.0)
Sleeping in the metaverse with a head-mounted display	76 (22.3)
Sleep duration <6 hours	110 (26.4)
Going to bed after 1 a.m.	272 (65.2)
Change in sleep quality since starting metaverse use	
Improved	29 (7.0)
Decreased	75 (18.0)
No change	313 (75.1)

Table 6 (other findings) reveals that 141 (33.8%) had low health literacy and 283 (67.9%) expressed a desire to consult medical professionals in the metaverse.

**Table 6:** Health literacy and willingness to access medical consultations in the metaverse.

	*n* (%)
Health literacy	
Low health literacy	141 (33.8)
Medium health literacy	155 (37.2)
High health literacy	121 (29.0)
Desire to consult with a medical professional in the metaverse	283 (67.9)

## Discussion

We surveyed the health status of 417 metaverse users. Approximately 70% of respondents were in their 20s and 30s, approximately 70% were male, and 96.2% resided in Japan. VRChat was the most commonly used platform, mainly accessed via head-mounted displays and PCs. Many participants engaged with the metaverse regularly, with over half spending three or more hours per session.

Approximately 40% of users reported health problems, and 20% to 30% experienced reduced physical activity, physical fitness, or pain after beginning metaverse use. While some users maintained exercise habits in the metaverse, over 30% engaged in little or no moderate-to-vigorous physical activity. Most participants had BMIs in the normal range, yet many exhibited unbalanced dietary habits. While mental health improvements were reported by many, roughly 20% experienced poor sleep quality. Notably, approximately 70% of the participants expressed interest in consulting with medical professionals within the metaverse.

Despite a relatively high number of users reporting exercise habits, a certain number of participants had prolonged sedentary time and low physical activity levels. For reference, the average sedentary time among Japanese adults aged 20 to 40 is 5.1 to 5.6 hours per day, and 24% to 31% sit for over 8 hours daily [16]. Prolonged sedentary behavior is associated with muscle loss and increased risk of lifestyle-related diseases [17]. Many participants exceeded national averages in sedentary time, suggesting that such behavior, perhaps owing to desk work and metaverse use, may have become routine. In contrast, 97 participants (23.3%) reported having exercise habits in the metaverse. Virtual reality exercise, including exercise in the metaverse, is enjoyable and enhances positive emotional attitudes toward exercise [18]. Providing places in the metaverse that motivate exercise has also been reported to promote behavioral change related to exercise and increase physical activity levels [19]. Exercise in the metaverse often involves mutual communication, which may make these activities easy to continue.

Approximately 20% of the respondents reported a decline in physical function after starting metaverse use. While physical inactivity related to metaverse usage may contribute, this may also reflect the effects of the COVID-19 pandemic, as many participants began using the metaverse during this time. Notably, 36% of participants reported little to no moderate-to-vigorous physical activity. According to the World Health Organization, adults should engage in at least 150-300 minutes of moderate-intensity or 75-150 minutes of vigorous-intensity aerobic activity weekly [20]; participants in this study may be at increased health risk due to insufficient physical activity. However, the Physical Activity Vital Sign used in this study relies on self-reported questionnaires, potentially underestimating actual physical activity levels compared to device-based measurements.

Compared to the National Health and Nutrition Survey in Japan, which indicated that the percentage of Japanese adults aged 20-40 with regular exercise habits was 23.5% to 26.5% for men and 14.5% to 16.9% for women [21], this study’s participants reported a relatively high prevalence of such habits. Some users even chose to stand during metaverse use, suggesting that they may be partially compensating for physical inactivity through metaverse-based movement. Still, approximately 30% of the participants experienced physical pain after beginning metaverse use, likely related to head-mounted displays, which are also known to strain the neck and shoulders [22]. Improvements in comfort and weight reduction of these devices may be necessary for prolonged use.

In the current survey, vision deterioration was reported by 18.2% of participants. While this may be related to metaverse use, it is also consistent with increased screen time during the COVID-19 pandemic [23]. Additionally, many users had unbalanced diets. A lack of dietary diversity has been linked to adverse health outcomes, including reduced physical function and mortality [24]. Previous studies have associated prolonged gaming with poor dietary habits and meal skipping [25], particularly among young adults living alone. Creating spaces in the metaverse for sharing nutrition-related information may promote better eating habits.

In this study, 20% of the respondents increased their alcohol consumption after beginning metaverse use. Virtual drinking parties may contribute, along with the ease of *logging off* and sleeping afterward without facing real-world consequences. Restricted real-world sensory input in the metaverse may also delay awareness of intoxication.

Sleep quality worsened for 18% of users, with many reporting later bedtimes. Previous studies have linked extended gaming with shorter sleep duration and poor sleep quality [26]. Interestingly, 22% of participants reported sleeping while wearing a head-mounted display. While this may help some fall asleep faster, it may also reduce sleep quality [27]. More research is needed to understand how such practices impact rest.

In this survey, 46% of participants reported having mental health issues; however, 37% experienced improvements in their mental health after starting metaverse use. Prior studies suggest that frequent users may experience higher levels of anxiety, depression, and loneliness [28]. Conversely, virtual social interaction can also reduce loneliness and promote a sense of belonging, thereby reducing anxiety and stress and improving well-being [28]. Recently, psychological and social support has been provided in the metaverse for mental health issues [29]. The use of avatars may increase familiarity, making communication easier, facilitating consultation about problems, and reducing loneliness. The psychological impact of the metaverse appears highly individualized, depending on user characteristics and usage patterns. Therefore, future research and practice should leverage these benefits while carefully addressing the potential risks associated with metaverse engagement. In the future, research and utilization of the metaverse to promote mental health benefits will be required.

To improve health behaviors within the metaverse, strategies adapted to the metaverse environment can be useful. In this survey, many participants were in the *contemplation* stage of behavioral change, and around 30% of them had low health literacy. These results indicate a need for interventions that support decision-making and create environments conducive to health choices.

Exercise programs provided in the metaverse have the potential to increase physical activity levels, improve motivation to maintain exercise habits, and enhance exercise self-efficacy. Offering a variety of activities that users can choose from may promote sustained engagement. Likewise, personalized digital nutritional guidance is effective in promoting healthier eating habits [30], and dietary education events and recipe sharing in the metaverse could serve as a framework to encourage autonomous choice-making behavior. For mental health, virtual interventions have shown promise in alleviating social anxiety and depression [31]. Given many users’ interest in consulting with medical professionals, offering anonymous support in the metaverse may be beneficial. Regarding sleep, online sleep hygiene programs could be adapted to the metaverse, including features like bedtime reminders and calming environments. For users with low health literacy, the metaverse may serve as a psychologically safe place for promoting healthy decision-making. In the future, intervention designs should offer flexible, diverse options tailored to users, especially those in earlier stages of behavioral change.

Interventions targeting metaverse users offer various benefits. In the metaverse, interventions can be conducted remotely and simultaneously with large numbers of people. Additionally, targeting metaverse users allows direct outreach to individuals with specific health risks. Effectively reaching those with low mobility, who are difficult to reach through traditional approaches, holds significant public health implications. Psychological approaches based on the unique characteristics of the metaverse can be applied to promote behavioral changes in health-related behaviors. For example, interactive communication and gamification have been reported to improve adherence to virtual reality exercises [32]. Metaverse communities enable support for healthy behaviors through powerful interactive communication. Some exercise events currently held in the metaverse introduce gamification, such as rewarding participants with avatars for the accumulation of participation points. Enhancing the body awareness of avatars during exercise may also increase motivation for exercise in the metaverse. In virtual spaces, the appearance of avatars can influence behavior within the metaverse, a phenomenon known as the Proteus effect [33], which may be useful for promoting behavioral change in the metaverse. Designing metaverse worlds, avatars, and events tailored to healthy behaviors may be effective in promoting healthy behavioral change.

Although this survey was a meaningful attempt to provide insight into the health status of the metaverse users, there are several limitations. First, participant recruitment through social media may have introduced selection bias, as those interested in health or active in-metaverse exercise events may have been more inclined to participate, while users with poorer health may have avoided the survey. Meanwhile, a large-scale lifestyle survey [34] of over 2,000 metaverse users included data from 1,547 Japanese respondents (77%); the VR platform most commonly used was VRChat, similar to our study, and the age, sex, frequency of metaverse use, and average play time per session were also similar to our survey. Thus, we believe that our data sampling is not significantly biased. In addition, as a cross-sectional study, we cannot establish causal relationships between metaverse use and health status. It remains unclear whether metaverse use leads to inactivity or if less active individuals are more drawn to the metaverse. In the future, longitudinal studies are needed to assess changes over time. Furthermore, the survey was conducted primarily in Japanese and involved mostly Japanese residents, which limits its generalizability to other populations. In addition, we did not conduct subgroup analyses by metaverse platform due to concerns about discrimination, though platform-specific differences likely exist. Survivor bias should also be considered, as only users who continued metaverse use were surveyed. Data from those who discontinued due to adverse effects were not included. Lastly, there is also the issue of measurement bias. Some participants may not have answered truthfully about themselves. Many of the scales used to measure mental health have not been properly validated. Concerns remain about the uncertainty of mental health measurements.

## Conclusions

This study surveyed 417 metaverse users to assess their health status. While various health issues, such as insufficient physical activity, unbalanced diets, and poor sleep, were observed, the presence of users who stand, exercise, and report mental health benefits within the metaverse was also evident. In addition, many participants were in the “contemplation” stage of health behavior change and lacked the ability to make and act on healthy behaviors independently, underscoring the need for systems that support autonomous health-related decision-making. The high demand for consultation with medical professionals highlights the need to implement health support systems within the metaverse. Rather than being a source of health problems, the metaverse holds potential as a platform for promoting behavioral change-if supported by appropriate design and interventions. Future efforts should focus on developing mechanisms that combine enjoyment and health promotion, making the metaverse a space that supports well-being as well as social interaction.
